# Anticandidal activity of *Clinopodium chilense* essential oil

**DOI:** 10.3389/fphar.2025.1634250

**Published:** 2025-07-10

**Authors:** Iván Montenegro, Constanza Villarroel, Evelyn Muñoz, Karel Mena-Ulecia, Valentina Silva, Alejandro Madrid

**Affiliations:** ^1^ Center of Interdisciplinary Biomedical and Engineering Research for Health (MEDING), Escuela de Obstetricia y Puericultura, Facultad de Medicina, Universidad de Valparaíso, Viña del Mar, Chile; ^2^ Laboratorio de Productos Naturales y Síntesis Orgánica (LPNSO), Facultad de Ciencias Naturales y Exactas, Universidad de Playa Ancha, Valparaíso, Chile; ^3^ Millennium Nucleus Bioproducts, Genomics and Environmental Microbiology (BioGEM), Valparaíso, Chile; ^4^ Departamento de Ciencias Biológicas y Químicas, Facultad de Recursos Naturales, Universidad Católica de Temuco, Temuco, Chile; ^5^ Núcleo de Investigación en Bioproductos y Materiales Avanzados (BIOMA), Vicerectoría de Investigación y Postgtado, Universidad Católica de Temuco, Temuco, Chile

**Keywords:** Clinopodium chilense, essential oil, pulegone, thymol, *Candida* spp

## Abstract

The antifungal activity of the essential oil of *Clinopodium chilense* (Benth.) Govaerts was investigated against several strains of *Candida* spp. including clinical isolates and reference strains. Antifungal efficacy was evaluated by determining minimum inhibitory concentration (MIC) and minimum fungicidal concentration (MFC). The chemical composition of the essential oil, characterized by gas chromatography-mass spectrometry (GC/MS), revealed pulegone (18.5%), thymol (11.0%), and isomenthone (10.0%) as the primary constituents. *Clinopodium chilense* essential oil (EO) demonstrated broad-spectrum anticandidal activity against all tested *Candida* spp., with MIC values ranging from 16 to 64 μg/mL and MFC values from 16 to 128 μg/mL. The EO exhibited potent fungicidal activity (MFC/MIC ratio ≤2) against several strains, notably *Candida tropicalis* (MIC and MFC of 16 μg/mL), and also showed efficacy against *C. guilliermondii* and *C. lusitaniae*. Among the major components, thymol generally displayed the lowest MIC values (32–64 μg/mL). Molecular docking studies further suggested thymol as a key contributor to the oil’s antifungal effect, showing strong binding affinities to *C. albicans* virulence proteins Als9-2 and the enzyme CYP51. Significantly, the essential oil outperformed amphotericin B against all tested clinical isolates. Overall, *C. chilense* EO exhibits significant fungistatic and fungicidal activity against pathogenic *Candida* species without affecting normal cell viability. These findings, supported by chemical characterization and *in silico* analysis of its major components like thymol, highlight its potential as a source of novel natural antifungal agents.

## 1 Introduction

A global health problem of growing relevance is candidiasis, an opportunistic fungal infection caused by *Candida* species, with *C*. *albicans* being the most common etiological agent (Macias-Paz et al., 2023). This infection can manifest itself in various forms, from superficial mucocutaneous infections to life-threatening invasive systemic infections, particularly in immunocompromised individuals (Lionakis et al., 2023). Conventional treatment of candidiasis is based on the use of synthetic antifungals, such as azoles and echinocandins; however, the increasing prevalence of strains resistant to these drugs (Fisher et al., 2022) has prompted the search for more effective and safer alternative therapies. In this context, essential oils (EOs) have emerged as a promising alternative due to their antimicrobial and antifungal properties and their lower probability of inducing resistance (Sobhy et al., 2025). EOs are complex mixtures of volatile compounds produced by various plants, and their antimicrobial activity has been attributed to the synergy between their components (Ben Miri, 2025). Specifically, EOs derived from plants of the Lamiaceae family have demonstrated remarkable antifungal activity against *Candida* spp. (Potente et al., 2020).

The Lamiaceae family, also known as the mint family, is one of the largest and most economically important plant families with a global distribution (Panda et al., 2022). In Chile, this family is represented by diverse genera and species, including the genus *Clinopodium* (Morales, 2018). Although some species of the genus *Clinopodium*, such as *Clinopodium gilliesii*, have been studied and the presence of bioactive compounds, including monoterpenes and sesquiterpenes, has been reported in their EO composition (Carvajal et al., 2017), the potential of the EO from the Chilean endemic species *Clinopodium chilense* remains unexplored.

*Clinopodium chilense* (Benth.) Govaerts is an evergreen aromatic shrub reaching up to 1.5 m in height. Commonly known as “oreganillo” or “menta de árbol” (tree mint), it is distinguished by its mint-like aroma (Baeza, 1930) and its drought tolerance, behaving as a partially deciduous shrub (Montenegro et al., 1979). Its geographic distribution in Chile spans from the Coquimbo region to the Araucanía region (Muñoz et al., 1981). Over time, this species has been classified under various synonyms, including *Gardoquia gilliesii* Graham, *G*. *chilensis* Benth., *Satureja chilensis* (Benth.) Briq., and *Satureja gilliesii* (Graham) Briq (Morales, 2018). The ethnobotanical value of *C. chilense* lies in its traditional uses. Its leaves and flowers, both dried and ground, are used as a condiment similar to oregano. Furthermore, a mild and pleasant-tasting infusion, similar to tea, is made from the dried leaves and flowers (Cordero et al., 2017; Cordero et al., 2022). In folk medicine, the infusion of “menta de árbol” has been traditionally used to relieve stomach aches, treat indigestion, and as a stimulant (Muñoz et al., 1981). Additionally, its use as an antiseptic in washing pig intestines intended for sausage making has been documented (Cordero et al., 2022). *C. chilense* is also employed in the restoration of degraded soils and as an ornamental plant in floral gardens (Riedemann et al., 2014). From a chemical perspective, only two studies have been reported on the dichloromethane extract of this species. One study, focusing on the leaves, identified the monoterpenes acetylsaturejol and isoacetylsaturejol, in addition to the sesquiterpenes T-cadinol and (−)-Cadin-4-en-l-ol (Labbe et al., 1993). The other study isolated seven diterpenoids from the flowering aerial parts: one labdane, three isopimaranes, and three rearranged isopimaranes (Labbé et al., 1994). In both studies, the toxicity of the compounds was evaluated using the *Artemia salina* assay (Labbe et al., 1993; Labbé et al., 1994). However, no prior studies have investigated the composition of the essential oil of *C*. *chilense*, nor its potential biological activity, including its antifungal capacity against *Candida* species. This lack of information represents a significant gap in knowledge, especially considering the potential of essential oils as sources of novel antifungal agents.

Therefore, this research aims to provide the first comprehensive characterization of the essential oil of *C. chilense*, analyzing its chemical composition and evaluating the antifungal activity of the oil and its major components against five strains of *Candida* spp. The results of this study could not only reveal the antifungal potential of this endemic Chilean species but also contribute to the discovery of novel bioactive compounds with therapeutic applications in the treatment of candidiasis.

## 2 Materials and methods

### 2.1 Chemistry

All reagents, pulegone, thymol, isomenthone, amphotericin B, itraconazole and fluconazole were purchased from Sigma-Aldrich Co. (St. Louis, MO, United States), GIBCO BRL Life Technologies (Grand Island, NY, United States), and Santa Cruz Biotechnology (Santa Cruz, CA, United States).

### 2.2 Plant material

*C*. *chilense* was collected from Viña del Mar, Valparaiso Region, Central Chile (S: 33°02′38″, W: 71°30′04″) during the spring in October 2020. Mr. Patricio Novoa confirmed species authenticity, and a voucher specimen (Cch-1020) was deposited at the Natural Products and Organic Synthesis Laboratory of Universidad de Playa Ancha, Valparaíso, Chile.

### 2.3 Essential oil and analysis

The EO was extracted from the aerial parts (leaves and branches) of *C*. *chilense* (500 g) by hydrodistillation carried out using a Clevenger-type apparatus for 4 h. The EO was collected and dried over anhydrous sodium sulfate and stored in sealed brown vials at 4°C. Subsequently, *C*. *chilense* EO was diluted with dichloromethane, and 1 μL of the sample was analyzed using a GC-MS/MS (Hewlett-Packard GC/MS 6890 coupled to a Hewlett-Packard 5973 mass-selective detector) operating in EI mode at 70 eV, equipped with a splitless injector (250°C). The transfer line temperature was 200°C. Helium was used as a carrier gas at a flow rate of 1.2 mL/min, and the capillary column used was a HP-5 ms (60 m × 0.25 mm i.d., film thickness 0.25 μm). The temperature program was 40°C (5 min) to 300°C (5 min) at a rate of 5°C/min. Volatile compounds were identified by comparing their mass spectra, obtained using the Thermo Xcalibur 3.1.66.10 program, with the NIST 2020 library database, and by comparison of their retention index with those reported in the literature. The retention indices were determined under the same operating conditions in relation to a homologous n-alkanes series (C_8_–C_36_).

### 2.4 Antifungal activity

#### 2.4.1 Fungal strains

The antifungal activity of the EO and its main components was evaluated against reference strains and clinical isolates of *Candida*. The reference strains *C. albicans* (ATCC 7978) and *C. parapsilosis* (ATCC 22019) were used, as well as the clinical isolates *C. albicans* 10,935, *C. dubliniensis* 3240, *Candida glabrata* 10,912, *C. guilliermondii* 12,204, *C. lusitaniae* 2305, and *Candida tropicalis* 9841. The clinical isolates were obtained from patients of the Base Hospital of Valdivia, Los Ríos Region, Chile. After identification, the microorganisms were included in the pathogenic fungal collection (Bioassay Laboratory of University of Valparaíso). They are maintained in Sabouraud Dextrose Broth (SDB) with glycerol at −80°C according to established protocols (Madrid et al., 2012).

#### 2.4.2 Fungal growth (MIC and MFC)

The antifungal susceptibility testing was performed in accordance with the guidelines in (CLSI, 2008) reference protocols M27-A3 for yeasts, were used to determine the minimal inhibitory concentration (MIC) and minimal fungicidal concentration (MFC) of the EO and their major compounds (pulegone, thymol, isomenthone), as described previously (Madrid et al., 2025). Briefly, cultures of all yeast were placed on Sabouraud Dextrose Agar (SDA) and incubated for 24–72 h at temperature 37°C. Colonies of this culture were suspended in sterile 0.85% NaCl and the inoculum was standardized according to the scale of 0.5 McFarland (1–5 × 10^6^ CFU/mL). The antifungal assay was conducted in 96-well plates. Yeast strains were prepared in sterile water and diluted in RPMI 1640 medium (excluding the sterility control). stock solutions of essential oil and pure compounds were diluted two-fold with RPMI from 256 to 0.03 μg/mL (final volume = 100 µL), with a final DMSO concentration of 1% w/v. A volume of 100 µL of inoculum suspension was added to each well with the exception of the sterility control where sterile water was added to the well instead. Reference antifungal drugs, amphotericin B, itraconazole, and fluconazole, were used as positive controls, whereas DMSO served as the negative control.

The MIC determination was conducted with approximately 0.5-2.5 × 10^3^ CFU/mL of the microorganism in each well. Following a 24–48 h incubation at 37°C, the absorbance of the plates was measured at 540 nm using a VERSA Max microplate reader (Molecular Devices, Sunnyvale, CA, United States) for spectrophotometric analysis. The MIC endpoint was calculated as the lowest concentration giving rise to an inhibition of growth equal to or greater than 80% of that of the growth control (MIC), similar to the visual endpoint criterion recommended by the CLSI. After determining the MIC, the minimum fungicidal concentration (MFC) was determined as a sub-culture of 2 μL of each of the wells that showed no growth and were incubated for 72 h at 37°C. The lowest concentration with no visible growth was defined as MFC indicating 99.5% death of the original inoculum. All experiments were performed in triplicate and repeated three times for reproducibility.

### 2.5 Docking studies

#### 2.5.1 Construction of ligands

To carry out the molecular docking studies, a computer equipped with an Intel^®^ Core™ i7 processor and running the Windows 10 Pro 64-bit operating system was used. The three-dimensional models of the ligands were built using UCSF Chimera 1.18 software. Polar hydrogens were added to each ligand, Gasteiger charges were assigned, and energy minimization was performed using the General AMBER Force Field (GAFF).

#### 2.5.2 Molecular docking

The three-dimensional crystallographic structures of the N-terminal domain of Als9-2 from *C*. *albicans* in complex with the gamma peptide of human fibrinogen (PDB ID: 2Y7L; Resolution: 1.49 Å) (Manzano et al., 2024) and Sterol 14-alpha demethylase (CYP51) from *C*. *albicans* (PDB ID: 5TZ1; Resolution: 2.0 Å) (Jabba and Jordt, 2019) were retrieved from the RCSB Protein Data Bank (PDB) (https://www.rcsb.org/) and used as docking targets. AutoDock Tools (version 1.5.6) was employed to prepare these protein structures, which involved the removal of water molecules, metal atoms, co-crystallized ligands, and other non-covalently bound substances. Kollman charges and both polar and non-polar hydrogens were added, and the target files were saved in the corresponding pdbqt format. The grid coordinates were set at −1.943 Å (X), −11.223 Å (Y), and 29.743 Å (Z) for PDB 2Y7L, and 67.837 Å (X), 36.744 Å (Y), and 39.072 Å (Z) for PDB 5TZ1. The grid box dimensions were 20 points (X), 20 points (Y), and 20 points (Z) for both targets. The search parameters included 50 runs with a maximum of 25,000,000 evaluations per ligand. The RMSD threshold for multiple clusters was set to <1.6 Å. The results were ranked based on binding energy and potential conformations. The lowest binding energy and the most probable conformation were selected for further analysis. Finally, Discovery Studio Visualizer (version 21.1.0.20298) was used to generate two-dimensional and three-dimensional images of the most stable selected conformation.

#### 2.5.3 Prediction of physicochemical and toxicological parameters

For the obtaining of pharmacokinetic and toxicological parameters, the chemical structures of the analyzed compounds in SMILES format were used on the SwissAdme platform (http://www.swissadme.ch/) and ADMETlab 2.0 (https://ai-druglab.smu.edu/).

#### 2.5.4 Molecular dynamic simulations

For the molecular dynamics simulations, the best poses from the docking experiments between the proteins Als9-2 (PDBid: 2Y7L) (Salgado et al., 2011) and CYP51 PDBid: 5TZ1) (Hargrove et al., 2017) from *Candida albicans* and the compounds pulegone, thymol and isomenthone were used as a starting point. These best poses were chosen taking into account the most negative binding energies and the lowest RMSD (Bell and Zhang, 2019; Velázquez-Libera et al., 2020) parameter values. Proteins were prepared by adding hydrogen atoms at physiological pH (pH = 7.4) using the UCSF Chimera software version 1.14 (Goddard et al., 2018; Pettersen et al., 2021; Meng et al., 2023). The force field used was CHARMM36 (Vanommeslaeghe and MacKerell, 2012; Gutiérrez et al., 2016; Huang et al., 2017) for proteins, and parameters for organic molecules were obtained from the SwissParam web server (Zoete et al., 2011). The proteins-ligand complexes were placed into a rectangular water box of 15 × 15 × 15 Å3 centered on the mass center of each ligand, using the TIP3P water model (Boonstra et al., 2016). All the complexes were submitted to 5000 steps for energy minimization using the conjugated gradient methodology at a temperature of 298.15 K using the weak coupling algorithm (Berendsen et al., 1984). The Van der Waals cutoff was fixed to 12 Å, under the NPT ensemble (Number of particles, Pressure and Temperature constant). The complexes studied were submitted a 1.0 fs time step under the velocity Verlet algorithm; 2.0 ns of equilibration and 100 ns of molecular dynamics simulation using the NAMD 2.13 software package (Bhandarkar et al., 2003; Phillips et al., 2005; Acun et al., 2018; Phillips et al., 2020).

### 2.6 Cytotoxicity

#### 2.6.1 Cell lines and culture conditions

The cytotoxicity of *C. chilense* EO was evaluated in two non-tumorigenic human cell lines: CCD 841 CoN (colon epithelial cells) and HEK-293T (human embryonic kidney). All tested cell lines were obtained from the AmericanType Culture Collection (Rockville, MD, United States). The different cell lines were maintained as monolayers in a culture medium (HAM-F10 + DMEM, 1:1) supplemented with 10% fetal bovine serum, as well as antibiotics (0.01 mg/mL streptomycin and 0.005 mg/mL penicillin). The cells were incubated at 37 °C in a humidified 5% CO_2_ atmosphere.

#### 2.6.2 Cell viability assay

To determine the effect on cell viability of *C. chilense* EO, the sulforhodamine B (SRB)assay was used to measure the number of viable cells after each treatment (Silva et al., 2022). Cells were seeded in 96-well plates at 3 × 10^3^ cells/well in 100 μL of culture medium and treated in triplicate with increasing concentrations of essential oil (0.625–100 μg/mL) for 72 h at 37°C and 5% CO_2_. The cells which received only the medium containing 0.1% DMSO served as the control group. After each incubation, the cells were fixed with trichloroacetic acid, then washed by immersion in distilled water and stained with 50 μL/well of SRB, as well as 0.1% (w/v) in 1% (v/v) acetic acid at room temperature for 30 min. The dye is solubilized with 150 μL/well of 10 mM Tris Base, and the absorbance is then measured at a wavelength of 540 nm in a microplate reader. 5-fluorouracil (5-FU) was used as positive control. Values shown are the mean +SD of three independent experiments in triplicate. Finally, Sigma Plot software (Systat Software, San Jose, CA, United States) was used to calculate the IC_50_ value.

### 2.7 Statistical analysis

The data were reported as the mean values ±standard deviation (SD). Due to non-parametric data, a Kruskal–Wallis ANOVA was used with a confidence level of 95% with the STATISTICA 7.0 program.

## 3 Results and discussion

### 3.1 EO composition

The EO of *C*. *chilense* was obtained with a yield of 1.9% (v/w). The EO of *C*. *chilense* fresh leaves is composed mainly by oxygenated monoterpenes (44.6%), followed by oxygenated sesquiterpenes (20.3%), monoterpene esters (11.7%), phenols (11.0%) and hydrocarbon sesquiterpenes (8.6%) (Table 1).

**TABLE 1:** Essential oil composition of *C*. *chilense*.

No	RT (min)	Components	% Area^a^	RI^a^	RL^b^	Identification
1	14.56	cis-sabinol	1.5	1103	1105	RL, MS
2	14.95	Menthone	2.1	1131	1131	RL, MS, Co
3	15.03	Neoisopulegol	2.2	1133	1134	RL, MS
4	15.28	Isomenthone	10.0	1139	1139	RL, MS, Co
5	15.35	Borneol	0.5	1149	1149	RL, MS, Co
6	15.66	4-terpineol	3.9	1159	1160	RL, MS, Co
7	16.82	α-terpineol	3.4	1197	1198	RL, MS, Co
8	17.46	Pulegone	18.5	1211	1211	RL, MS, Co
9	18.38	isomenthyl acetate	2.3	1250	1251	RL, MS
10	18.63	Isopulegol acetate	3.2	1260	1260	RL, MS, Co
11	18.72	bornyl acetate	5.3	1276	1277	RL, MS, Co
12	18.95	Thymol	11.0	1278	1278	RL, MS, Co
13	20.51	Ascaridole	0.5	1279	1280	RL, MS
14	21.15	α-copaene	0.6	1334	1334	RL, MS
15	21.41	beta-bourbonene	3.1	1338	1339	RL, MS
16	22.83	(R)-(+)-citronellic acid	2.0	1341	1341	RL, MS
17	23.02	Menthofurolactone	0.9	1354	1353	RL, MS
18	24.68	γ-amorphene	2.5	1479	1480	RL, MS
19	24.96	cis-calamenene	2.4	1521	1521	RL, MS
20	26.26	(−)-spathulenol	1.5	1574	1576	RL, MS
21	26.39	caryophyllene oxide	1.5	1580	1581	RL, MS, Co
22	27.12	Epicubenol	7.9	1611	1613	RL, MS
23	27.70	t-cadinol	8.4	1631	1630	RL, MS
24	28.01	4(15),5,10(14)-germacratrien-1-ol	1.0	1680	1680	RL, MS
		Total identified	96.2			
		Oxygenated monoterpenes	44.6			
		Oxygenated sesquiterpenes	20.3			
		Monoterpene esters	11.7			
		Phenols	11.0			
		Hydrocarbon sesquiterpenes	8.6			

^a^ Experimental retention index for non-polar column.

^b^ bibliographic retention index for non-polar column, MS: mass spectra.

**FIGURE 1:**
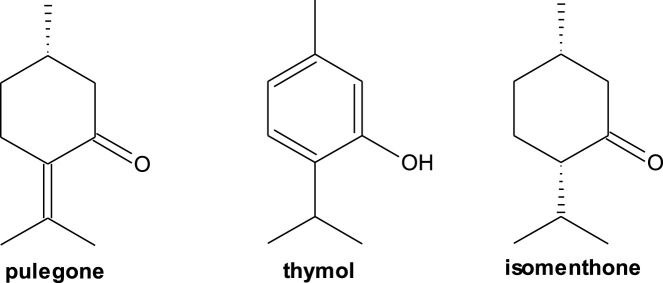
Major compounds present in *C. chilense* EO.

Twenty-four compounds were identified in the EO of *C*. *chilense*, which corresponded to 96.2% of the total oil analyzed, and the main components were pulegone (18.5%), thymol (11.0%), and isomenthone (10.0%) (Figure 1).

This is the first study on *C. chilense* EO, a plant often confused with *Clinopodium gilliesii* due to shared scientific nomenclature and similar uses, especially in culinary applications. However, a crucial difference lies in the concentration of pulegone, the major compound in both. While *C. gilliesii* exhibits a pulegone content of 93.8% (Carvajal et al., 2017), *C. chilense* contains only 18.5%, suggesting a lower risk associated its consumption of the latter. This difference is critical because *C. chilense* is often confused with *C. gilliesii* due to similar nomenclature and uses, especially in culinary applications. The lower pulegone concentration, coupled with the presence of thymol and isomenthone (monoterpenes also found in edible plants like thyme and peppermint), suggests a more favorable safety profile for C. chilense (de Sousa Barros et al., 2015; Salehi et al., 2018). In contrast, the essential oil of *Clinopodium nubigenum* from Ecuador, traditionally used to treat colds and stomach aches, exhibited a high pulegone content (72.8%), with 3,7-dimethyl-1,6-octadien-3-ol (7.0%) and cis-isopulegone (4.7%) as secondary components, and demonstrated antimicrobial activity against *C*. *albicans* (Gilardoni et al., 2011). On the other hand, analysis of four wild *Clinopodium* species, traditionally used in folk medicine from countries of the former Yugoslavia, revealed a predominance of oxygenated monoterpenes: *C. thymifolium* (35.2% pulegone), *C. serpyllifolium* (13.0% pulegone, 38.7% piperitenone oxide), *C. dalmaticum* (27.3% pulegone, 41.7% piperitenone), and *C. pulegium* (22.9% pulegone, 34.1% piperitenone oxide) (Dunkić et al., 2017). It is important to note that not all *Clinopodium* species contain pulegone as the primary component. The essential oil of *Clinopodium gracile* collected in China lacks pulegone in its composition, instead exhibiting germacrene D (20.6%), nootkatone (8.2%), and morillol (7.7%) as major compounds, yet it still demonstrated larvicidal activity against *Aedes albopictus* (Chen et al., 2013). These variations emphasize the necessity of carefully characterizing each species and its geographical origin to determine their potential uses and safety.

### 3.2 Antifungal activity

The antifungal activity (MIC and MFC) of EO and their main compounds is presented in Table 2.

**TABLE 2:** Minimum inhibitory concentration (MIC) and Minimum Fungicidal Concentration (MFC) of *C*. *chilense* EO and and their main compounds against *Candida* spp. MIC and MFC values are expressed in μg/mL.

Sample	A		B		C		D		E		F		G	
	MIC	MFC	MIC	MFC	MIC	MFC	MIC	MFC	MIC	MFC	MIC	MFC	MIC	MFC
EO	32	64	64	128	16	16	32	32	64	128	64	64	64	128
1	128	128	128	256	128	256	64	64	64	128	64	128	128	128
2	64	64	32	64	64	128	32	64	32	32	32	64	64	64
3	128	128	128	128	64	128	64	64	128	128	128	128	128	128
C1	1.0	2.0	0.5	1.0	32	64	64	128	128	256	64	128	128	256
C2	0.5	1.0	1.0	2.0	0.5	1.0	2.0	4.0	2.0	4.0	0.5	1.0	32	64
C3	0.5	1.0	1.0	1.0	0.5	1.0	4.0	8.0	4.0	8.0	2.0	4.0	8	16
DMSO	I		I		I		I		I		I		I	

A, *C. albicans* (ATCC, 7978); B, *C. parasilopsis* (ATCC, 22019); C, *C. tropicalis* (9841); D, *C. lusitaniae* (2305); E, *C. albicans* (10,935); F, *C. guilliermondii* (12,204); G, *C. glabrata* (10,912); 1, pulegone; 2, thymol; 3, isomenthone; C1, amphotericin b; C2, fluconazole; C3, itraconazole. Each value represents the mean of three experiments (*p* < 0.05), performed in quadruplicate. I: inactive.

Table 2 summarizes the activity observed at 48 h for *C*. *chilense* EO and its major compounds, specifically those found in abundances exceeding 10%. In terms of results, *C. lusitaniae* proved to be the most sensitive strain to the different treatments, with *Candida tropicalis* following closely behind. In contrast, *Candida glabrata* proved to be the least sensitive species to the various treatments. This observation is consistent with previous research, including a study by Deravi et al. (2021) which also identified *C. glabrata* as a less susceptible species in clinical isolates of vulvovaginal candidiasis.

Analyzing the relationship between the MFC (Minimum Fungicide Concentration) and MIC (Minimum Inhibitory Concentration) values is essential for a thorough understanding of the mode of action of each treatment. The ratio MFC/MIC was calculated in order to determine if the essential oil or its major compounds had a fungistatic (MFC/MIC ≥4) or fungicidal (MFC/MIC ≤4) activity (Boubrik et al., 2025). In this regard, the *C. chilense* EO demonstrated broad antifungal activity against all tested *Candida* spp., with MIC and MFC values ranging from 16 to 128 μg/mL. Notably, the oil exhibited fungicidal activity (MFC/MIC = 1) against *C. guilliermondii*, *C. lusitaniae*, and *C. tropicalis*, with particularly potent activity observed against *C. tropicalis* at the lowest tested concentration of 16 μg/mL. All compounds exhibited antifungal activity, but the *C. chilense* EO demonstrated a remarkable ability to inhibit the growth of all strains at significantly lower concentrations compared to most individual compounds. Studies indicate that pulegone, one of the components of the EO, can inhibit the growth of various *Candida* strains, including *C. albicans*. Some research also demonstrates that pulegone can affect biofilm formation, a mechanism that helps *Candida* spp. resist antifungal drugs (Alam and Ahmad Khan, 2021). These results suggest an antifungal potential of pulegone against the analyzed yeast species. Notably, it exhibited a favorable MFC/MIC ratio of 1 against *C. lusitaniae* at a concentration of 64 μg/mL. However, for the remaining species, the MFC/MIC ratio was 2, requiring higher concentrations to achieve fungicidal effects. Concerning thymol, while it exhibited lower MIC values than pulegone, it demonstrated a superior fungicidal potential with an MFC/MIC ratio of 1 against four strains (*C. albicans* (ATCC 7978), *C. lusitaniae*, *C. albicans* (10,935), and *C. glabrata*). Notably, this group included *C. albicans* ATCC and a clinical isolate, with its potency being greater against the clinical isolate at concentrations two times lower than those required for the ATCC strain. For the remaining strains tested, the MFC/MIC ratio was 2. This phenol is well-known for its ability to disrupt the cell membrane and combat biofilm formation (Wang et al., 2017). However, at high concentrations or with prolonged exposure, thymol can be irritating to the skin and mucous membranes (Gavliakova et al., 2013). On the other hand, isomenthone generally inhibited all tested strains at a concentration of 128 μg/mL, exhibiting a fungicidal MFC/MIC ratio of 1, indicating its fungicidal potency at that concentration. The exception was *C. lusitaniae*, which, similarly to pulegone, was inhibited at a lower concentration of 64 μg/mL. This terpenketone along with other compounds such as menthone, has been shown to inhibit the growth of various fungi. It has been specifically reported that this type of monoterpene exhibits activity against *Aspergillus niger* (Moghtader, 2013). These findings are consistent with the known mechanism of action of terpenoids, the main components of the essential oil, which destabilize cell membranes, increase their permeability, and consequently disrupt and destroy microorganisms (Gonçalves et al., 2007). Further highlighting the potential of this EO, while amphotericin B proved more effective against ATCC strains, the EO outperformed it against all clinical isolates. While the EO exhibited higher MIC values than the evaluated azoles, it demonstrated superior fungicidal potential, with an MFC/MIC ratio of 1, compared to the value of 2 observed for both azoles across all strains. This MFC/MIC value aligns with that reported by Costa et al. (2017) for *C. albicans* and *C. parapsilopsis*. Although fluconazole is prototypically considered a fungistatic agent, our study demonstrated fungicidal activity against diverse *Candida* spp., with an observed MFC/MIC ratio of 2. This finding, far from being anomalous, is consistent with the growing body of evidence that azole activity is conditional. Our results align with those of Maheronnaghsh et al. (2016), who also reported low MFC/MIC ratios in isolates from cancer patients. Mechanistically, it has been shown that fluconazole, at the high concentrations used to determine the MFC, induces a programmed cell death (apoptosis) cascade in *C. albicans* (Lee and Lee, 2018), the most prevalent species. Furthermore, the fungicidal activity of this azole is not only dose-dependent but also influenced by the microenvironment. For example, Moosa et al. (2004) demonstrated *in vitro* that fluconazole becomes fungicidal under the acidic conditions that simulate the vaginal environment. This conditional activity has clear clinical relevance, as it has been validated *in vivo* by Sobel et al. (2003), who documented the potent efficacy of fluconazole in treating *C. albicans* vaginitis (Akins, 2005). Therefore, in the face of the conditional efficacy of conventional drugs, these results suggest a promising future for *C. chilense* EO as a potential alternative against pathogenic yeasts, thus opening new phytopharmacological possibilities.

### 3.3 The molecular docking study

A molecular docking study assessed the major compounds of *C. chilense* EO against two key receptors involved in *Candida* spp. pathogenicity; the results are presented in Table 3.

**TABLE 3:** Binding energies and interactions of the main compounds present in *Clinopodium chilense* oil with the N-terminal domain of *Candida albicans* adhesin Als9-2 (PDB ID: 2Y7L) and sterol 14-alpha demethylase (CYP51) of *Candida albicans*.

Compound	Enzyme (PDB code)	Binding energy (kcal/mol)	Key interactions
Pulegone (1)	2Y7L	−6.38	Val22; Pro29; Arg171; Lys59; Ile172
	5TZ1	−7.02	Hse327; Phe233; Pro230; Phe380; Met508; Leu121; Leu373
Thymol (2)	2Y7L	−6.98	Val22; Arg171; Pro29; Tyr297; Glu27
	5TZ1	−7.86	Ser378; Phe380; Leu376; Pro230; Phe233
Isomenthone (3)	2Y7L	−6.57	Val23; Pro30; Tyr298; Ile173; Lys60
	5TZ1	−6.62	Ser334; His333; Leu332; Ser334; Pro186; Met464; Leu77
Amphotericin B	2Y7L	−8.95	Trp295; Trp296; ser181; Tyr271; Arg172; Lys60; Phe59
	5TZ1	−10.38	His333; Tyr74; Phe419; Ile427; Gly428; Arg337
Fluconazole	2Y7L	−7.97	Ser170; Trp295; Glu27; Gly296; Ile172; Leu179; Pro29 Arg171; Val22; Trp294
	5TZ1	−6.90	Leu121; Pro230; Met508; Tyr64; Pro375; Leu376; Arg377
Itraconazole	2Y7L	−9.96	Tyr21,Val22; Arg171; Ile167; Val161; Ser169; Trp223 Thr168; Tyr23; Asn224; Ser159
	5TZ1	−10.51	Ala61; Phe58; Leu87; Leu121; Leu376; Ile304; Pro230 Ile471; Met508; Pro462; Gly472

The interactions detailed in Table 3 were assessed against key receptors critical for *Candida* spp. virulence. One such receptor, Als9-2, is an adhesin of *C*. *albicans* that facilitates fungal attachment to host cells by interacting with fibrinogen. This interaction contributes to biofilm formation and virulence in mucosal, systemic, and device-related infections. Sterol 14-alpha demethylase (CYP51) is a key enzyme in ergosterol biosynthesis, essential for fungal cell membrane integrity. Inhibition of CYP51 disrupts the membrane, enhancing the effectiveness of antifungal treatments. Both represent important therapeutic targets against *C. albicans* (Lim et al., 2025; Mesileya et al., 2025).

DMSO was used as the solvent control in the molecular docking experiments. As expected, no relevant binding affinities or specific interactions were observed. Although recent evidence indicates that DMSO may influence certain physical or kinetic properties—such as medium viscosity or protein stability—under specific conditions, it does not directly interact with the active site nor act as a competitive ligand (Tunçer and Gurbanov, 2023; Feoli et al., 2024).

The lowest binding energies were obtained for the compounds used as positive controls—amphotericin B, fluconazole, and itraconazole—with values of −8.95, −7.97, and −9.96 kcal/mol, respectively, for protein 2Y7L, and −10.38, −6.90, and −10.51 kcal/mol, respectively, for protein 5TZ1. These results indicate that the major compounds of *C. chilense* EO have binding affinity for two key proteins associated with the pathogenicity of *C*. *albicans*: the Als9-2 adhesin (PDB ID: 2Y7L) and the sterol 14α-demethylase enzyme (CYP51, PDB ID: 5TZ1). As expected, the reference compounds exhibited the best binding energies at both receptors, validating the docking methodology used.

Among the natural compounds evaluated, thymol (compound **2**) showed the strongest binding affinities toward both proteins, outperforming pulegone (**1**) and isomenthone (**3**). This greater affinity suggests that thymol may significantly contribute to the antifungal activity of the essential oil. Several studies have demonstrated that thymol possesses potent antifungal activity against *Candida albicans*, including fluconazole-resistant strains. For instance, thymol has been shown to inhibit biofilm formation and reduce fungal cell viability. Moreover, when combined with conventional antifungals such as fluconazole, thymol exhibits synergistic effects that enhance treatment efficacy (Jafri and Ahmad, 2020).

Studies on *Cryptococcus neoformans*, another human fungal pathogen, have investigated the mode of action of thymol. Results indicate that thymol disrupts ergosterol biosynthesis—an essential component of the fungal cell membrane—by modulating the expression of genes involved in this pathway, such as *ERG1*, *ERG11*, and *HMG1*. Additionally, thymol induces intracellular calcium homeostasis imbalance and interferes with protein glycosylation, processes that are critical for fungal cell viability (Jung et al., 2021).

Pulegone and isomenthone also showed interactions with both molecular targets, but with higher (i.e., less favorable) binding energies, suggesting lower affinity and, likely, a reduced contribution to the overall antifungal activity. A study by Miri et al. (2022) reported that the essential oil of *Mentha pulegium*, containing 74.81% pulegone, exhibited significant antimicrobial activity against several microorganisms, including *C. albicans*. These findings suggest that pulegone may be responsible for the observed antifungal activity, although its precise mechanisms of action remain unknown.

**FIGURE 2:**
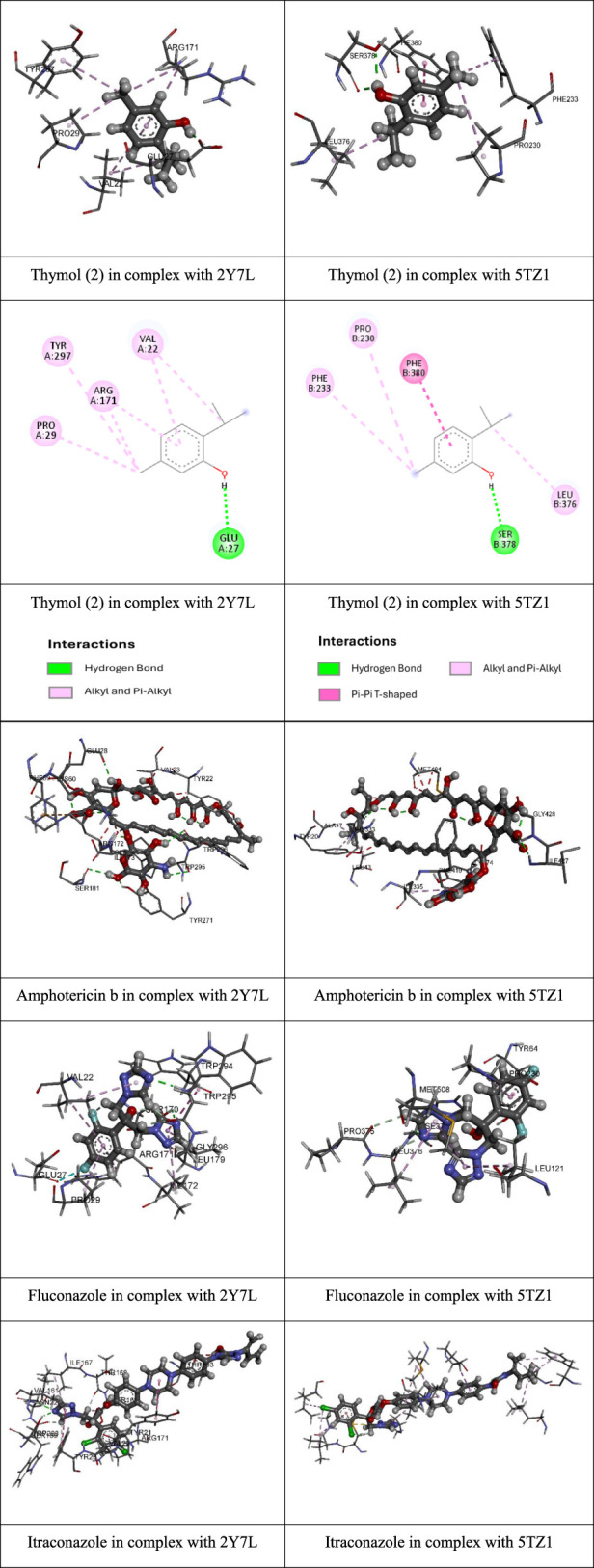
2D and 3D images representing the most stable complexes between thymol and the target proteins.

The compounds tested exhibited binding energies against 2Y7L ranging from −6.38 kcal/mol (pulegone, compound **1**) to −6.98 kcal/mol (thymol, compound **2**). For the 5TZ1 protein, binding energies ranged from −6.62 kcal/mol (isomenthone, compound **3**) to −7.86 kcal/mol (thymol, compound **2**). In both cases, thymol demonstrated the strongest binding affinities. Among the three compounds evaluated against different *Candida* strains, compound 2 (thymol) showed the highest antifungal activity. Molecular docking studies suggest that this compound binds efficiently to both target proteins (Figure 2).

In the case of the Als9-2 adhesin (PDB ID: 2Y7L), thymol formed a hydrogen bond with residue Glu27 at a distance of 1.87 Å, along with multiple alkyl interactions involving residues Val22, Arg171, Pro29, and Tyr297. These interactions suggest a stable binding to the N-terminal domain of the adhesin, potentially interfering with *C. albicans* adhesion mechanisms to host cells.

Regarding the sterol 14α-demethylase (CYP51, PDB ID: 5TZ1), thymol also exhibited a favorable interaction pattern, forming two conventional hydrogen bonds with residue Ser378 (at 2.00 and 2.26 Å), a T-shaped π–π interaction with Phe380 (at 5.08 Å), and additional alkyl interactions with residues Leu376, Pro230, and Phe233. These findings indicate that thymol may interfere with ergosterol biosynthesis, a key function targeted by azole antifungal agents.

The analysis of the physicochemical and toxicological parameters of the major compounds found in the essential oil of *Clinopodium chilense* is presented in Tables 4, 5. These data support the evaluation of their viability as candidates with pharmacological potential.

**TABLE 4:** Pharmacokinetics parameters.

Compound	Log *P*_o/w_	Water solubility	GI	BBB permeant	P-gp substrate	Log *K*_p_ [cm/s]
Pulegone (1)	2.71	Poorly soluble	High	Yes	No	−5.04
Thymol (2)	2.82	Poorly soluble	High	Yes	No	−4.87
Isomenthone (3)	2.65	Poorly soluble	High	Yes	No	−5.08

Log *P*_o/w_: Logarithm of the octanol/water partition coefficient (P); GI, absorption: Gastrointestinal absorption; BBB, permeant: Permeability of the blood-brain barrier; P-gp substrate: Transport by P-glycoprotein; Log *K*_p_: Logarithm of the cutaneous permeability coefficient.

**TABLE 5:** Prediction of toxicological parameters.

Compound	hERG Blockers %	Ames %	DILI %	LD_50_ -log (mol/kg)	Lipinski ruler
Pulegone (1)	33.39	42.56	44.66	1.84	Yes; 0 violation
Thymol (2)	33.56	41.24	42.9	1.99	Yes; 0 violation
Isomenthone (3)	34.35	52.88	46.06	2.01	Yes; 0 violation

hERG, Blockers %: The human ether-a-go-go related gene; Ames %: Ames test for mutagenicity. DILI %: Drug-induced liver injury. LD_50_: Lethal Dose 50.

The three compounds analyzed exhibited similar Log P values, indicating comparable lipophilicity profiles. This property favors permeability across biological membranes without compromising aqueous solubility (Chloe, 2024). Moreover, a high predicted gastrointestinal absorption and the ability to cross the blood–brain barrier was observed, suggesting good oral bioavailability and the potential to exert effects on the central nervous system, depending on the therapeutic application.

None of the compounds showed affinity for P-glycoproteins, thus reducing the risk of active efflux and enhancing their potential for intracellular accumulation. The skin permeability coefficient (Kp) was similar for all three compounds, with values ranging from −4.87 to −5.09 cm/s, suggesting limited dermal absorption—an important consideration for the development of topical formulations.

Regarding toxicological parameters, all compounds showed approximately 30% inhibition of the hERG channel, indicating a moderate but not alarming cardiotoxicity risk. Ames (mutagenicity) and DILI (drug-induced liver injury) tests yielded values below 50%, except for isomenthone, which showed an Ames result of 52.88%. This finding may warrant further evaluation in future studies. Additionally, the lethal dose 50 (LD_50_) values indicated relatively low acute toxicity, ranging from 1.84 mol/kg for pulegone to 2.01 mol/kg for isomenthone.

Although pulegone has been identified as a potential carcinogen in some toxicological studies involving prolonged high-dose exposure in rodents (Jabba and Jordt, 2019; Voigt, et al., 2024), these findings do not necessarily preclude its use in therapeutic contexts. The carcinogenic effects observed are highly dose-dependent and were associated with sustained oral intake, not short-term or localized administration. In our case, pulegone is considered for antifungal use, which could involve topical or controlled-release formulations that minimize systemic absorption and toxicological risks. Furthermore, many clinically approved drugs carry similar concerns but remain in use when the therapeutic benefits outweigh potential adverse effects. As such, the potential of pulegone as an antifungal agent remains valid, though further preclinical toxicological assessment will be necessary to ensure its safe medical application.

**FIGURE 3:**
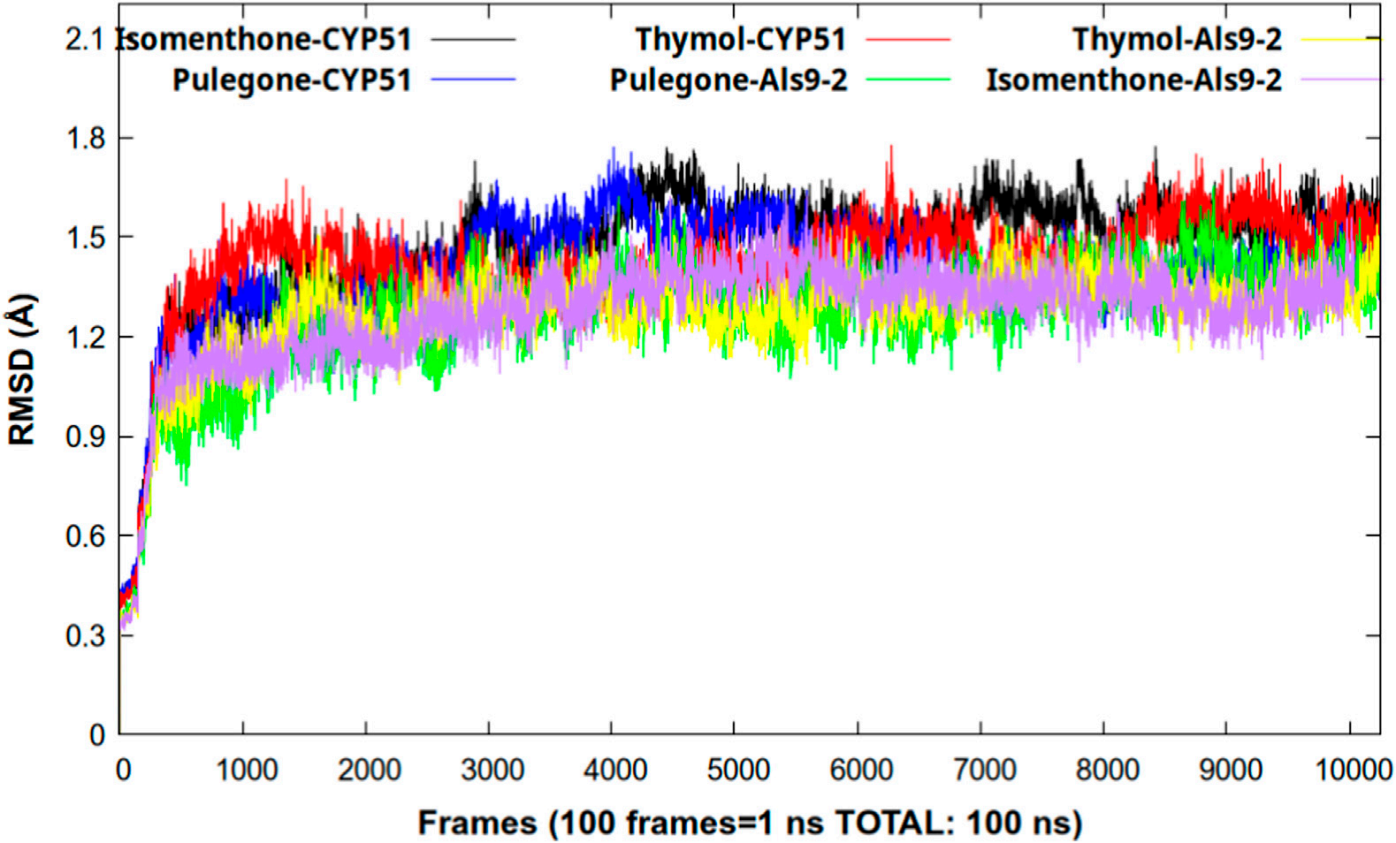
Graphical representation of the RMSD parameter during the simulation time.

Finally, it is worth noting that all three compounds meet the criteria set by Lipinski’s Rule of Five, supporting their potential as bioactive molecules with pharmacokinetic properties suitable for oral administration. To validate their effectiveness at the molecular level, molecular dynamics simulations were performed, providing information on the dynamic behavior of the complexes, as well as details at the molecular level during the simulation period. The first parameter to analyze is the RMSD, which provides a criterion for stability throughout the simulation period. As can be seen in Figure 3, all the complexes studied stabilized after 2 ns of simulation time. It is important to note that this parameter remained low throughout the 100 ns of the trajectory, with RMSD values below 2 Å, indicating high stability of the ligands in the active sites of both proteins. This suggests that the compounds could be good inhibitors of Als9-2 and CYP51 proteins.

From the graph shown in Figure 3, we can see that the lowest RMSD values were found in the complexes formed by the Als9-2 protein with pulegone and thymol, with values of 1.2663 ± 0.1628 Å and 1.2656 ± 0.1843 Å, respectively. These values indicate no significant differences between these complexes and the others, so it is necessary to analyze other variables obtained from the trajectory.

**FIGURE 4:**
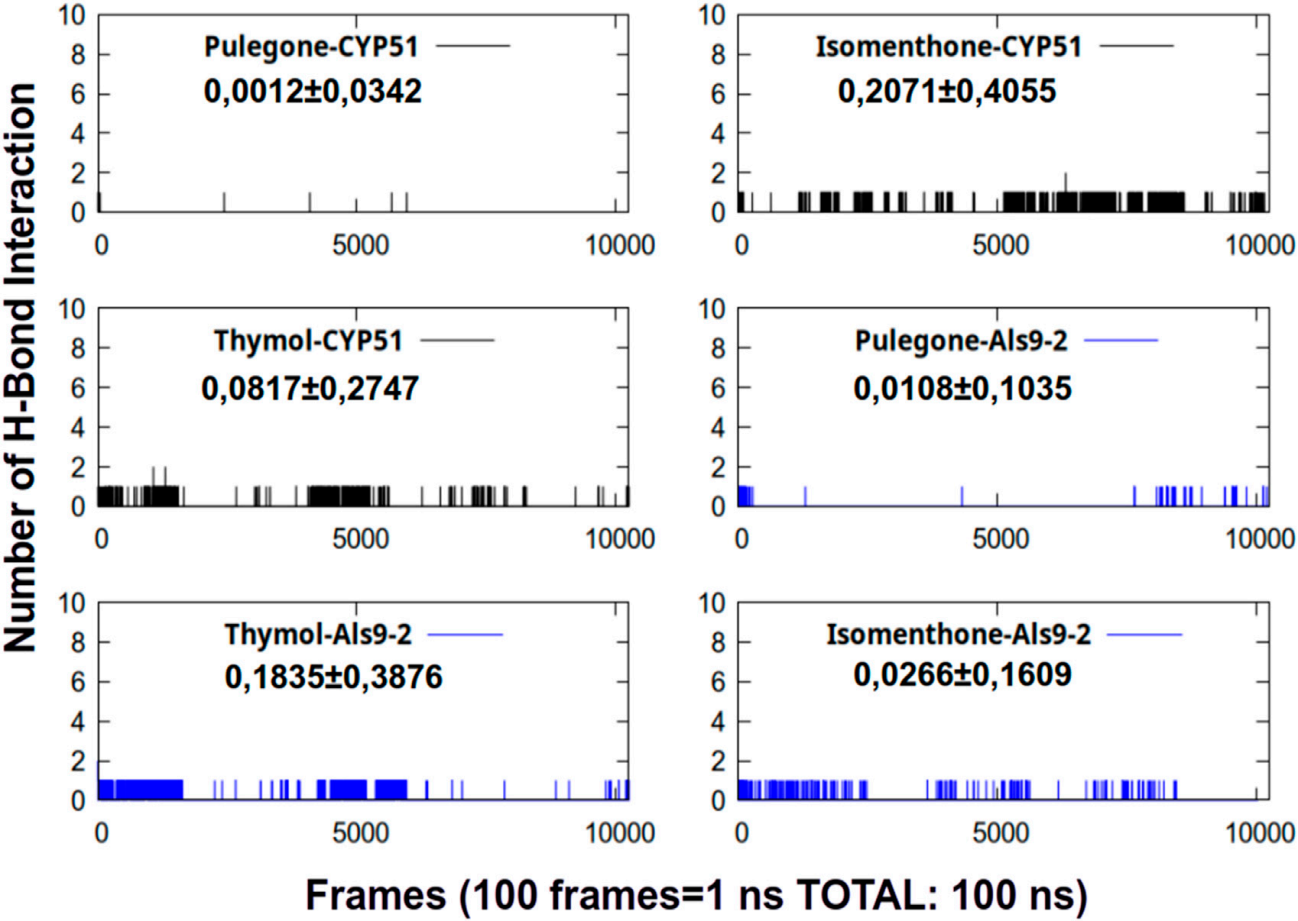
Number of hydrogen between the complexes studied during 100 ns of simulation time.

Hydrogen bonding interactions between the ligand and the protein confer stability to the complex over time. In our case, and as seen in Figure 4, this parameter’s behavior was weak during the 100 ns of simulation time.

The largest number of hydrogen bonding interactions (H-Bond) were found in the complexes formed by Isomenthone-CYP51 and thymol-Asl9-2. It is important to note that the standard deviation of this parameter was much higher than the mean for all simulated systems, indicating that this type of interaction was not stable over time. Based on the results obtained for this parameter, we can conclude that hydrogen bonding interactions do not explain the stability of the complexes studied.

To corroborate the behavior of the hydrogen bond interactions obtained during the simulation, we will analyze the interaction occupancy term. This term calculates the percentage of hydrogen bond interactions that remained below 3 Å during the simulation. This parameter is shown in Table 6.

**TABLE 6:** H-bond occupancies analysis from 100 ns of simulation time.

Complex	H-bond interaction	Occupancy (%)
Isomenthone-CIP51	TYR88-Side	12.53%
	THR78-Side	7.19%
Pulegone-CIP51	TYR64-Side	0.07%
	GLN66-Side	0.02%
Thymol-CIP51	ARG469-Main	2.87%
	THR311-Side	2.29%
Isomenthone-Als9-2	ILE172-Main	1.37%
	LYS59-Side	1.18%
Pulegone-Als9-2	ILE172-Main	0.44%
	ASN299-Side	0.20%
Thymol-Als9-2	GLU27-Side	9.02%
	GLU277-Side	8.28%

**FIGURE 5:**
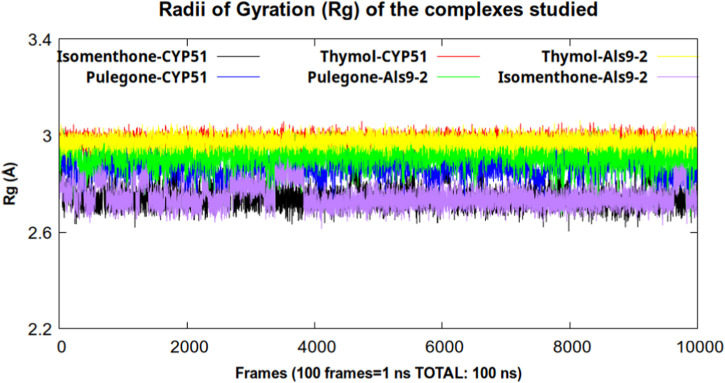
Radius of Gyration (Rg) of the Cα atoms in the complexes studied during 100 ns of simulation time.

For a hydrogen bond interaction to be considered stable during the trajectory, the occupancy percentage should be above 50% (Tsujimura et al., 2025). As can be seen in Table 6, in none of the complexes did the occupancy percentage exceed 15%. This corroborates the previously stated finding that these interactions were not stable during the 100 ns of simulation time, which does not explain the stability found in the RMSD parameter. Therefore, it is necessary to analyze other parameters such as radius of gyration (Rg) (Figure 5).

The variable radius of gyration is defined as the root mean square distance of the mass center of atom or collection atoms from a common mass centers and shows us the degree of compaction that a system presents during the trajectory (Lobanov et al., 2008).

As we can see in Figure 5, the lowest values for this parameter were found in the complexes formed by isomenthone and pulegone with CYP51, indicating greater compaction of these systems and, consequently, greater stability. The complex formed by isomenthone-CYP51 had the highest number of hydrogen bonds calculated and the highest occupancy, indicating that this compound could be a good inhibitor of the CYP51 enzyme.

### 3.4 Cytotoxicity assay

Due to the side effects and resistance that can arise from synthetic drugs used to treat *Candida* infections, both cutaneous and systemic, the search for alternatives in natural products, such as essential oils, has intensified for effective therapies against candidiasis or candidemia. In this context, it is crucial to know the cytotoxic effect that these products may have on healthy cells, in order to ensure their safe application in therapies for these pathologies. Consequently, in this study, the cytotoxic effect of *C. chilensis* EO on two non-tumorigenic human cell lines was investigated using an SRB assay (Table 7).

**TABLE 7:** Half-maximal inhibitory concentration (IC_50_) values of *C. chilensis* EO on two non-tumorigenic human cell lines.

Sample	CCD 841 CoN IC_50_ (µg/mL)	HEK-293 IC_50_ (µg/mL)
*C. chilense* EO	93.45 ± 0.89	96.78 ± 0.24
5-FU	56.4 ± 0.31	18.32 ± 0.56

Data are reported as mean values ±SD of three different experiments with samples in triplicate. p < 0.05 vs control (ethanol-treated cells).

In contrast to the chemotherapeutic agent 5-fluorouracil (5-FU), the *C. chilense* EO exhibited low cytotoxicity against the non-tumorigenic cell line, as indicated by its IC_50_ value. This finding suggests a favorable safety profile for the EO, indicating a minimal impact on normal cells. This finding aligns with observations from other essential oils containing similar major components. As an example, a commercial *Mentha piperita* EO (commonly known as peppermint, a sterile hybrid of watermint and spearmint, widely cultivated for its menthol flavor) containing approximately 29.90% isomenthone, exhibited very low toxicity in human epidermal keratinocyte (HaCaT) cells up to a concentration of 400 μg/mL (Silva et al., 2022). Similarly*, Thymus caramanicus* Jalas (commonly called Iranian thyme, a thyme species endemic to Iran whose leaves have been used traditionally in Iranian medicine to treat various conditions and whose extracts have demonstrated antibacterial, neuroprotective, antinociceptive, and anticancer properties), its EO with 20.84% thymol, was non-toxic to the normal gingival HGF1-PI1 cell line but highly cytotoxic to KB (oral carcinoma) cells at low concentrations (Fekrazad et al., 2017). Furthermore, a study on *Minthostachys verticillata* EO (a wild plant from Argentina and Paraguay, commonly known as peperina or peppermint, primarily consumed as an infusion and to flavor mate and tereré, and used for medicinal purposes as a digestive, antispasmodic, and anti-inflammatory), which contains 60.0% pulegone, demonstrated no *in vitro* cytotoxicity or *in vivo* cytogenotoxicity at both low and high concentrations (Escobar et al., 2012). Notably, *Minthostachys mollis* (a wild plant commonly known as muña, peperina, poleo de Quito, tifo or tipo is a species of woody shrub, native to Peru, Bolivia, Colombia, Ecuador, Argentina and Venezuela), whose essential oil possesses the same three major components as the *C. chilense* EO and a similar pulegone concentration (12.4%, with 1.0% thymol and isomenthone), showed minimal cytotoxic effects on healthy HEK.230T cells at 687.60 ± 33.50 ug/mL (Benites et al., 2018). These observations support the existing literature, which indicates that thymol, pulegone, and isomenthone are effective inhibitors of cancer cells with a relative cytotoxic activity in normal cells (Da Rocha et al., 2012; Jung et al., 2012; Ezatpour et al., 2023). Moreover, numerous studies suggest that minor components can play a significant role in antibacterial activity, potentially through synergistic effects with other constituents (Miladinović et al., 2021). This synergistic effect is likely also relevant to cytotoxic activities in cells. Therefore, further investigation is warranted to evaluate the cytotoxic potential of these minor components, both individually and in various combinations, to fully elucidate their role(s) in toxicity against both tumor and non-tumor cells. Based on the results obtained and the consulted literature, *C. chilense* EO shows promising potential as a therapeutic agent. While the toxicity of its main components, such as pulegone, is acknowledged, the levels present in the oil would not reach the intake doses considered risky (Voigt et al., 2024), suggesting a negligible or very low health risk. Nevertheless, given that its application is envisioned in a dermatological solution and considering its apparent lack of cytotoxicity in non-tumor cells, the importance of a careful evaluation of the dosage and of further research to identify potential synergistic or antagonistic effects before its therapeutic use is emphasized.

## 4 Conclusion

This study demonstrated that *Clinopodium chilense* EO possesses broad-spectrum antifungal activity against *Candida*, even superior to amphotericin B in clinical isolates. Thymol, one of its main components, is the most promising due to its strong binding to key *Candida* virulence proteins and its favorable pharmacokinetic and toxicological properties. Their potential for oral use could be realized through formulations or encapsulations designed to minimize health risks. Therefore, *C. chilense* EO is presented as a source of bioactive molecules and a promising natural antifungal.

## Data Availability

The original contributions presented in the study are included in the article/supplementary material, further inquiries can be directed to the corresponding author.
